# Reviewer acknowledgement 2014

**DOI:** 10.1186/1755-8166-7-11

**Published:** 2014-02-14

**Authors:** Thomas Liehr, Henry Heng, Yuri Yurov

**Affiliations:** 1 Jena University Hospital, Institute of Human Genetics, Jena, Germany; 2 Center for Molecular Medicine and Genetics, Wayne State University School of Medicine, MI, USA; 3 National Research Center of Mental Health, Moscow, Russia

## Abstract

**Contributing reviewers:**

The Editors of *Molecular Cytogenetics* would like to thank all our reviewers who have contributed to the journal in volume 6 (2013).

## 

Dilek Aktas

Turkey

Mara Almeida

Brazil

Gilda Alves Brown

Brazil

Bonnet-Garnier Amélie

France

Nicholas Anagnou

Greece

Rouben Aroutiounian

Armenia

Egbert Bakker

Netherlands

Istvan Balogh

Hungary

John Barber

United Kingdom

Luca Bartolini

United States of America

Denise Batista

United States of America

David Betts

Ireland

Samarth Bhatt

United States of America

Lukrecija Brecevic

Croatia

Joern Bullerdiek

Germany

Josefa Cabrero

Spain

Juan Pedro Camacho

Spain

Jordi Camps

Spain

Zhong Chen

China

Sau Cheung

United States of America

Marcelo Cioffi

Brazil

Linda Cooley

United States of America

Zunyan Dai

United States of America

Josef Davidsson

Sweden

Edivaldo de Oliveira

Brazil

Jan Diblik

Czech Republic

Peter Duesberg

United States of America

Emel Ergul

Turkey

Susan Ernst

United States of America

Eliana Feldberg

Brazil

Amanda Figueiredo

Brazil

Manuel Garrido-Ramos

Spain

David Genevieve

France

Ad Geurts van Kessel

Netherlands

Gabriele Gillessen-Kaesbach

Germany

Rastko Golouh

Slovenia

Ute Grasshoff

Germany

Franz Gruber

Norway

Roberta Guilherme

Brazil

Oskar Haas

Austria

Claudia Haferlach

Germany

Julia Hentschel

Germany

Henrik Hjorth-Hansen

Norway

Galina Hovhannisyan

Armenia

Ivan Iourov

Russia

Vadym Kavsan

Ukraine

Elena Kirillova

Russia

Nadezda Kosyakova

Germany

Petr Kuglik

Czech Republic

Leslie Kulikowski

Brazil

Huabing Li

United States of America

Peining Li

United States of America

Caroline Mackie Ogilvie

United Kingdom

Chloe Mak

Hong Kong

Frantisek Marec

Czech Republic

Dom McMullan

United Kingdom

Maria Isabel Melaragno

Brazil

Clemens Mellink

Netherlands

Aurelia Meloni-Ehrig

United States of America

Martina Merkas

Croatia

Hasmik Mkrtchyan

Armenia

Wagner Molina

Brazil

Santiago Munne

United States of America

Veera Sekaran Nadarajan

Malaysia

Indrajit Nanda

Germany

Felix Niggli

Switzerland

Yi Ning

United States of America

Beata Nowakowska

Belgium

Nobuhiko Okamoto

Japan

Ettore Olmo

Italy

Moneeb Othman

Germany

George Patrinos

Greece

Lorenza Pecciarini

Italy

William Quinn

United States of America

Petr Ráb

Czech Republic

Laureana Rebordinos

Spain

Horacio Rivera

Mexico

Elena Rossi

Italy

Nikolai Rubtsov

Russia

Albert Schinzel

Switzerland

George Seidel

United States of America

Yiping Shen

United States of America

Frenny Sheth

India

Marsha Speevak

Canada

Pawel Stankiewicz

United States of America

Karoly Szuhai

Netherlands

Alan Thornhill

United Kingdom

Nathan Treff

United States of America

Vladimir Trifonov

Russia

Raymond Tubbs

United States of America

Hilde Van Esch

Belgium

Peter Vandenberghe

Belgium

François Vialard

France

Svetlana Vorsanova

Russia

Yang Wang

United States of America

Elzbieta Warchalowska-Sliwa

Poland

W. Steven Ward

United States of America

Jingly Weier

United States of America

Ulli Weier

United States of America

Anja Weise

Germany

Marjolein Willemsen

Netherlands

Jasen Wise

United States of America

